# Urban-rural and gender differential in depressive symptoms among elderly in India

**DOI:** 10.1016/j.dialog.2023.100114

**Published:** 2023-02-13

**Authors:** Shubham Kumar, Shekhar Chauhan, Ratna Patel, Manish Kumar, David Jean Simon

**Affiliations:** a Department of Mathematical Demography & Statistics, International Institute for Population Sciences, Mumbai, India; b Department of Population Policies and Programmes, International Institute for Population Sciences, Mumbai, India; c Department of Public Health and Mortality Studies, International Institute for Population Sciences, Mumbai, India; d Department of Sociology, Banaras Hindu University, India; e Paris 1 Pantheon Sorbonne University, France

**Keywords:** Depression, urban-rural differential, Gender differential, Decomposition analysis, India

## Abstract

**Background:**

To date, evidence remained inconclusive explaining rural-urban and male-female differential in depression. Unlike other previous research on the association of several risk factors with depressive symptoms among the elderly, this study focussed on the socio-economic status-related inequality in the prevalence of depression among the elderly along with focussing urban-rural and male-female gradients of depression among the elderly.

**Methods:**

This study used data from Longitudinal Ageing Study in India (LASI) Wave-I, 2017-18, survey. The outcome variable for this study was self-reported depression. Bivariate analysis was used to understand the prevalence by sociodemographic clusters. Fairlie decomposition analysis has been done to measures rural-urban inequalities for depression among older men and women.

**Results:**

Results found that around 22 percent of urban elderly and 17 percent of rural elderly reported depression. A higher proportion of female elderly (22.6% vs. 18.4%) reported depression than male elderly. Almost one in every five elderly (20.6%) reported depression in India. The results found that a higher percentage of women in rural and urban areas reported depression than their male counterparts. While examining SES-related inequality in the prevalence of depression, education was a significant factor explaining the SES-related inequality in the prevalence of depression among female elderly and not in male elderly.

**Conclusion:**

Given the large proportion of elderly reporting depression, this study highlights the need for improving health care services among the elderly. The increasing burden of depression in specific sub-populations also highlights the importance of understanding the broader consequences of depression among rural and female elderly.

## Highlights

• Around 22 percent of urban elderly and 17 percent of rural elderly reported depression.
• Almost one in every five elderly (20.6%) reported depression in India.
• SRH and IADL disability explained nearly 40 percent of the urban-rural inequality in the prevalence of depression among males.
• Increasing burden of depression highlights the importance of understanding the broader consequences of depression among rural and female elderly.

## 1 Introduction

The proportion of the elderly population in India has increased significantly over time. The older population has increased from 24.71 million in 1961 to 43.98 million in 1981, an increment of almost 78 percent [43]. Furthermore, it has increased from 43.98 million in 1981 to 104 million in 2011, an increment of almost 136 percent [33]. 104 million elderly in 2011 roughly translated into 8 percent of India’s total population, and the proportion of the elderly population is expected to rise by 20 percent by 2050 [39]. With such unprecedented growth in the elderly population over time, there is an escalating demand for geriatric healthcare in India. With an increase in life expectancy and decline in fertility levels, ageing phenomenon has transpired into a significant health problem. Depression among the elderly is one such concern that is rising off lately in India.

Depression is a leading cause of disability and is a significant contributor to the overall global burden of disease [3,17]. Globally, more than 264 million people of all ages suffer from depression [49]. Furthermore, depression has been ranked as the foremost contributor to non-fatal health loss, accounting for 7.5 percent of global years lived with disability (YLDs) and 2 percent of global disability-adjusted life years (DALYs) [47]. Depression can affect people from all backgrounds across the life course; however, it is more pronounced in older people [6]. The Elderly are more prone to psychological disturbances due to natural age-related decline in physiological functioning, leading to severe depression [48]. Currently, geriatric depression is a growing concern in India due to an increase in the proportion of the elderly population.

Previous studies have attributed depression among the elderly to certain risk factors. Studies have noted education [38,51], place of residence [38], marital status [18,51], age [38,45], gender [18,38,51], wealth index [38,51], physical activity [18], self-rated health [20], obesity [37], work status [38], functional impairment [38] as a risk factor of depression. Chronic medical illness is also one of the widely explored risk factor of depression [21,28]. However, impact of major depression are also said to affect chronic medical illness [22]. Nevertheless, there is a bidirectional relationship between depression and chronic medical illness. Despite the abundance of literature on risk factors associated with depression, there is no conclusive evidence on urban-rural and male-female gradients on depression among the elderly.

The prevalence of depression among the elderly has been increasing over time. With an ever-increasing proportion of the older population, it is expected that a higher number of older people than ever would fall for depression. It will not be an exaggeration to state that older people would be more vulnerable to depression in the coming days, given the current disease burden. So, there is a need to study the prevalence and factors associated with depression among the elderly in India. Furthermore, it is also important to examine the factors contributing to the socio-economic status (SES) related to inequality in the prevalence of depression among the elderly. To date, evidence remained inconclusive explaining rural-urban and male-female differential in depression. Certain studies noted higher depression among female elderly [12,34,53], while other few noted higher depressive symptoms among male elderly [32], and some others stated inconclusiveness while depicting gender differential in depression among elderly [52]. Therefore, this study examines the rural-urban and male-female gradient of depression among the elderly in India. Furthermore, this study decomposes the factors explaining the SES-related inequality in the prevalence of depression among the elderly.

## 2 Materials and methods

### 2.1 Data source

In this study, the first wave of the Longitudinal Ageing Study in India (LASI) was used as a data source which was conducted across all the 35 Indian states (exclude Sikkim) and union territories (UTs) in 2017-18. The study was funded by the Ministry of Health and Family Welfare (MoHFW), the Government of India, the National Institute on Aging (NIA), and the United Nations Population Fund, India (UNFPA). The nodal agencies of the survey were the International Institute for Population Sciences (IIPS), Harvard T.H. Chan School of Public Health (HSPH), and University of Southern California (USC), and several other national and international institutions.

The LASI is the latest survey that is well-positioned to assess the effect of changing policies on the behavioural outcomes of the elderly in India and considered the world’s most extensive and India’s first longitudinal study. The survey’s tools and protocols are harmonized with the Health and Retirement Study (HRS) in the United States and its sister surveys in Asia, Europe, and elsewhere.

The primary objective of the LASI is to contribute extensive scientific information on demographics, household economic status, chronic health conditions, symptom-based health status, functional health, mental health (cognition and depression), biomarkers, insurance, and healthcare utilization, family and social networks, social welfare programs, work and employment, retirement, satisfaction, and life expectancy.

The LASI survey instrument contains the Household Schedule (HH), Individual Schedule, biomarker survey, and community schedule. The HH schedule was surveyed with a selected vital informant in every household, where the individual and biomarker schedule was observed with each chosen respondent. Moreover, household schedule captured household roaster (Detailed information of household members), housing and environment, household consumption, household assets and debts, household income, and household health insurance. However, individual schedule included the demographic modules, the work, retirement and pension module, the health care access and utilization module which covered overall health in general, the family and social network module, the social welfare module, the experimental module, and biomarker and direct health examination module. Furthermore, biomarker survey measured the functional health, a lung function and a vision test, anthropometric measures etc. Lastly, community schedule collected information on population characteristics, infrastructure and common resources, the accessibility and availability of healthcare services, and the coverage of health and social welfare programs.

The LASI has applied a multistage stratified area probability cluster sampling to achieve the country’s representative sample. The survey has used a three-stage sampling design for rural areas and a four-stage sampling design for urban areas. The sampling frame has been adopted from the census of India (2011) to select sub-districts (Tehsils/Talukas) in the first stage of sampling. In the second stage, villages and wards were selected from sub-districts in rural and urban areas. The third stage involved selecting households from selected villages in rural areas; however, an additional stage was involved for urban areas. The list of census enumeration blocks (CEBs) was selected in the third stage in urban areas. To obtain the list of selected households in urban areas, mapping and listing of households have been done to reach out the final list of households from selected census enumeration blocks (CEBs).

The first wave of LASI included a sample of 72,250 individuals aged 45 years and above and their spouses; however, our study concerned 31,464 elderly aged 60 years and above, including 6,749 individuals aged 75 years above from 35 states and union territories.

### 2.2 Study Variables

#### 2.2.1 Outcome variable

The outcome variable for this study is “depression,” which was self-reported. The question has been asked, “During the last 12 months, was there ever a time when you felt sad, blue, or depressed for two weeks or more in a row” and responses recorded in dichotomous for as “yes” and “no.” Another outcome variable for this study is ‘thinking’ or ‘perception’ about depression. The question has been asked for thinking of depression as “did you lose interest in most things?”, “did you ever feel more tired out or low in energy than is usual for you?”, “did you lose your appetite?”, During the same two-week period, did you have a lot more trouble concentrating than usual? “people sometimes feel down on themselves, and no good or worthless, during those two weeks, did you feel this way?”, “did you think a lot about death – either your own, someone else’s, or death in general – during those two weeks?” and “did you have more trouble falling asleep than you usually do during those two weeks?”. All this question has been asked in the form of either ‘yes’ or ‘no.’

#### 2.2.2 Covariates

Covariates for this study considered as age (60-69 and 70 years and above); gender (male and female); marital status (currently married, never married, Divorced/Separated/Deserted/Widowhood); living arrangements (living alone, with spouse and with others); religion (Hindu, Muslim and others); education (No education, below primary, primary, secondary, and higher); place of residence (rural and urban); wealth index (poorest, poorer, middle, richer and richest); currently working (yes and no); self-rated health (poor and good); ); tobacco use (no and yes). Activity daily diving (ADL) has been constructed using five parameters: bathing, dressing, mobility, feeding, and toileting. Similarly, instrumental activity daily living (IADL) has created using seven parameters: preparing a hot meal (cooking and serving), shopping for groceries, making telephone calls, taking medications, doing work around the house or garden, managing money, such as paying bills and keeping track of expenses and getting around or finding an address in an unfamiliar place. Further, ADL and IADL disability classified into “severe,” “moderate,” and “no disability.”

### 2.3 Statistical measures

Data were analyzed using STATA version 16. Bivariate analysis was used to understand the prevalence and thinking of depression among sociodemographic clusters. The prevalence of depression has presented separately for men and women for rural and urban areas, respectively. Fairlie decomposition analysis has been done to measures rural-urban inequalities for depression among older men and women. Fairlie decomposition is a very simple technique used to estimate from a logit or probit model, first described by Fairlie in 1999 [9]. The decomposition results have been presented in terms of coefficient and percent contribution by a group of sociodemographic variables. The significance level is shown at 95% CI and 99% CI. The equation for fairlie decomposition can be written as:

$${Ӯ}^{U}-{\bar{Y}}^{R}=\left[\sum_{i=1}^{N^{U}}\frac{F\left(X_{i}^{U}{\hat{\beta }}^{U}\right)}{N^{U}}-\sum_{i=1}^{N^{R}}\frac{F(X_{i}^{R}{\hat{\beta }}^{R}}{N^{R}}\right]+\left[\sum_{i=1}^{N^{R}}\frac{F\left(X_{i}^{R}{\hat{\beta }}^{U}\right)}{N^{R}}-\sum_{i=1}^{N^{R}}\frac{F(X_{i}^{R}{\hat{\beta }}^{R}}{N^{R}}\right]$$

Where N^U^ and N^R^ is the sample size for urban and rural respectively, Ӯ^*U*^ and ${\bar{Y}}^{R}$ are the average probability of a binary outcome of interest for group urban and rural, F is the cumulative distribution function from the logistic distribution, distribution, *X*_*i*_^*R*^ and *X*_*i*_^*U*^ are the set of the average value of the independent variable and ${\hat{\beta }}^{U}$ and ${\hat{\beta }}^{R}$ are the coefficient estimates for the urban and rural respectively.

## 3 Results

Table 1 depicts the sociodemographic profile of the elderly population in India. Around three-fifths (58.5%) of the elderly belonged to the 60-69 years of age group, and the remaining (41.5%) belonged to 70+ years. The distribution of elderly by gender was nearly equal as 47.5 percent of the elderly were male, and the remaining 52.6 percent of elderly were female. Around three-fifths (62.1%) of the elderly were currently married. More than half of the elderly (56.5%) had no education, and only 4.1 percent of the elderly had higher education. Nearly 70 percent of the elderly belonged to rural areas, and the remaining 30 percent belonged to urban areas. Almost two-fifths (41.8%) of the elderly were working. Nearly 15 percent elderly reported poor self-rated health.

**Table 1:** Sociodemographic profile of elderly population aged 60 years and above in India.

	N	%
**Age**		
60–69	18,410	58.5
70+	13,054	41.5
**Gender**		
Male	14,931	47.5
Female	16,533	52.6
**Marital status**		
Currently married	19,536	62.1
Never married	225	0.7
Divorced/separated/deserted/widowhood	11,703	37.2
**Living arrangements**		
Living alone	1,787	5.7
With spouse	19,176	60.9
With others	10,501	33.4
**Religion**		
Hindu	25,871	82.2
Muslim	3,548	11.3
Others	2,045	6.5
**Education**		
No Education	17,782	56.5
Below primary	3,598	11.4
Primary	3,520	11.2
Secondary	5,285	16.8
Higher	1,278	4.1
**Place of residence**		
Rural	22,196	70.6
Urban	9,268	29.5
**Wealth Index**		
Poorest	6,829	21.7
Poorer	6,831	21.7
Middle	6,590	21.0
Richer	6,038	19.2
Richest	5,175	16.5
**Currently working**		
Yes	9,483	41.8
No	13,197	58.2
**Self-rated health**		
Poor	4,630	15.0
Good	26,181	85.0
**Tobacco use**		
No	18,665	59.8
Yes	12,539	40.2
Alcohol use		
Yes	4555	14.6
No	26655	85.4
**ADL disability**		
Severe ADL	999	3.2
Moderate ADL	6,045	19.3
No ADL	24,291	77.5
**IADL disability**		
Severe IADL	1,859	5.9
Moderate IADL	13,281	42.4
No IADL	16,164	51.6
**Total**	**31,464**	**100**

**Fig. 1:**
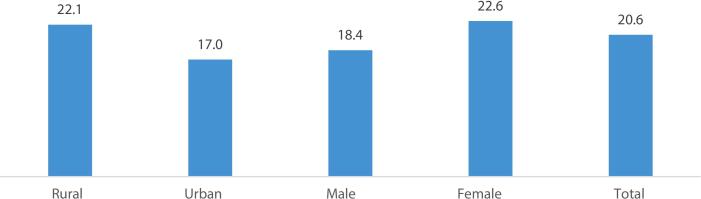
Prevalence of depression among elderly by place of residence and sex

Figure 1 shows the prevalence of depression among the elderly by their residence and sex. Results found that around 22 percent of urban elderly and 17 percent of rural elderly reported depression. A higher proportion of female elderly (22.6% vs. 18.4%) reported depression than male elderly. Almost one in every five elderly (20.6%) reported depression in India.

Table 2 depicts the gender-wise prevalence of self-reported depression among the elderly in rural and urban areas. The results found that a higher percentage of women in rural and urban areas reported depression than their male counterparts. Also, depression was more prevalent among the elderly in rural areas than in urban areas. Almost one-fifth (19.13%) of the rural men reported depression, and almost one-fourth (23.9%) of rural women reported depression. Similarly, around 15 percent of urban men reported depression, and 18 percent of urban women reported depression. A higher percentage of rural men with higher education (21.4% vs. 18.1%) reported depression than uneducated rural men, whereas a lower percentage of rural women with higher education (15.3% vs. 24%) reported depression than uneducated rural women. Results found that the reporting of depression was higher among the richest men and women from rural and urban areas than their poorest counterparts. Depression was lower among those who were currently working than their non-working counterparts. The prevalence of depression was higher among elderly men and women who reported poor self-rated health, severe ADL, and IADL disability.

**Table 2:** Gender-wise prevalence of self-reported depression among elderly in rural and urban in last 12 months for two or more weeks by sociodemographic characteristics.

	Rural Men			Rural Women			Urban Men			Urban Women		
	%	p-value	N	%	p-value	N	%	p-value	N	%	p-value	N
**Age**												
60–69	20.0	0.053	5,865	24.2	0.668	6,274	13.8	0.141	2,855	18.9	0.744	3,408
70+	17.8		4,212	23.4		4,374	14.9		2,166	16.6		2,310
**Marital status**												
Currently married	19.0	0.016	8,168	22.3	0.000	4,938	12.6	0.000	4,189	17.1	0.000	2,278
Never married	17.2		119	7.0		22	15.6		39	25.1		47
Divorced/separated/deserted/widowhood	19.7		1,790	25.3		5,688	22.9		793	18.5		3,393
**Living arrangements**												
Living alone	20.0	0.018	272	30.0	0.000	1,037	29.3	0.000	103	27.1	0.000	333
With spouse	18.9		8,052	22.3		4,825	12.5		4,102	16.8		2,229
With others	19.7		1,753	24.0		4,786	21.5		815	17.8		3,155
**Religion**												
Hindu	19.4	0.000	8,433	23.7	0.000	8,876	15.4	0.000	3,906	18.7	0.000	4,587
Muslim	17.4		993	24.0		1,028	11.5		829	16.8		780
Others	17.0		651	25.3		744	6.8		286	11.4		351
**Education**												
No Education	18.1	0.738	4,635	24.0	0.026	9,009	16.1	0.001	978	22.4	0.001	2,630
Below primary	21.4		1,592	25.3		739	15.9		563	18.7		714
Primary	20.8		1,539	22.4		533	16.9		682	17.9		827
Secondary	17.8		2,001	20.9		351	13.8		1,942	9.6		1,300
Higher	21.4		308	15.3		17	9.9		856	12.7		246
**Wealth Index**												
Poorest	18.7	0.013	2,034	22.8	0.230	2,411	15.1	0.632	1,129	23.0	0.008	1,268
Poorer	16.2		2,213	23.0		2,395	14.6		989	18.2		1,206
Middle	21.3		2,114	23.7		2,261	11.1		1,166	13.3		1,051
Richer	18.8		1,962	24.4		1,971	14.0		933	15.6		1,180
Richest	20.8		1,754	26.4		1,610	17.7		804	19.1		1,014
**Currently working**												
Yes	17.9	0.006	4,817	25.5	0.001	2,370	12.4	0.010	1,581	21.2	0.760	562
No	20.1		4,734	27.1		3,821	15.1		3,207	27.3		1,588
**Self-rated health**												
Poor	34.8	0.000	1,510	37.4	0.000	1,773	32.6	0.000	553	31.3	0.000	730
Good	16.7		8,361	21.7		8,651	12.8		4,360	16.4		4,873
**Tobacco use**												
Yes	16.8	0.000	3,433	23.6	0.028	7,786	12.4	0.000	2,723	16.8	0.270	4,909
No	20.5		6,569	24.6		2,809	18.1		2,216	27.1		759
**Alcohol use**												
Yes	20.0	0.264	2,864	19.6	0.146	349	18.6	0.000	1,224	29.1	0.921	49
No	19.5		6,947	24.6		10,040	14.0		3,639	18.4		5,514
**ADL disability**												
Severe ADL	33.0	0.000	285	28.2	0.000	390	33.4	0.000	140	27.4	0.000	180
Moderate ADL	26.6		1,781	31.9		2,351	26.3		704	26.7		1,155
No ADL	17.0		7,974	21.3		7,886	12.2		4,130	15.4		4,362
**IADL disability**												
Severe IADL	27.6	0.000	475	30.8	0.000	922	27.4	0.000	143	23.3	0.000	268
Moderate IADL	22.2		3,784	27.8		5,456	23.6		1,308	23.4		2,552
No IADL	16.5		5,770	17.2		4,229	11.1		3,522	12.8		2,874
**Total**	**19.13**		**10,077**	**23.9**		**10,648**	**14.9**		**5,021**	**18.1**		**5,718**

Note- The prevalence of depression calculated based on self-reporting asked for last 12 months for two or more weeks in a row.

**Fig. 2:**
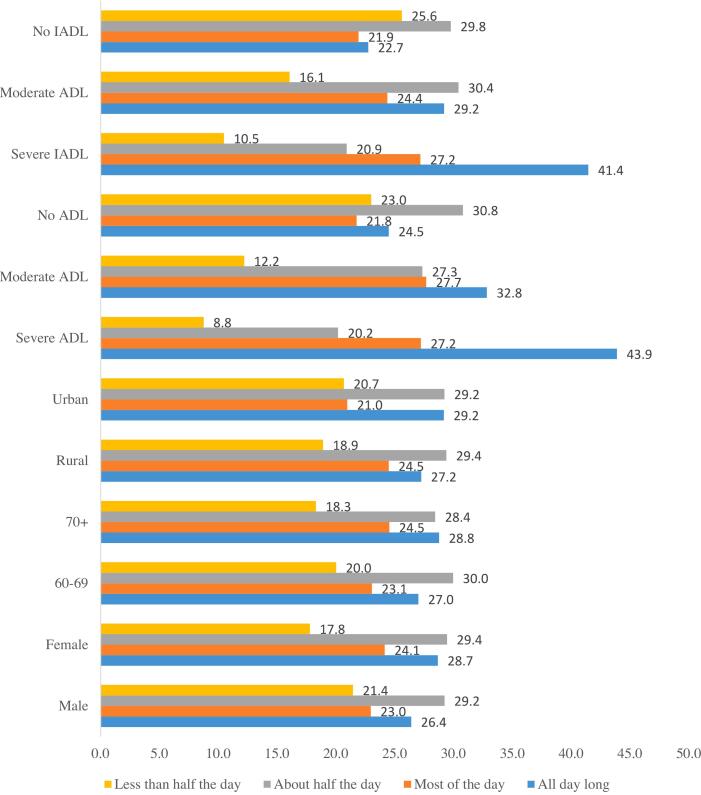
Experience of depression among elderly by age, gender, place of residence, living arrangements, ADL disability and IADl disability

Figure 2 depicts the experience of depression among the elderly by various characteristics. Results found that a higher percentage of those with severe ADL and IADL reported depression all day long. More than two-fifths of the elderly with severe ADL (43.87%) and severe IADL (41.44%) reported depression all day long. A higher percentage of elderly living alone reported depression all day long (29.66% vs. 27.01%) than elderly living with a spouse. Similarly, a higher percentage of urban elderly than rural elderly (29.16% vs. 27.24%) reported depression all day long. A higher percentage of female elderly than male elderly (28.65% vs. 26.39%) reported depression all day long.

**Fig. 3:**
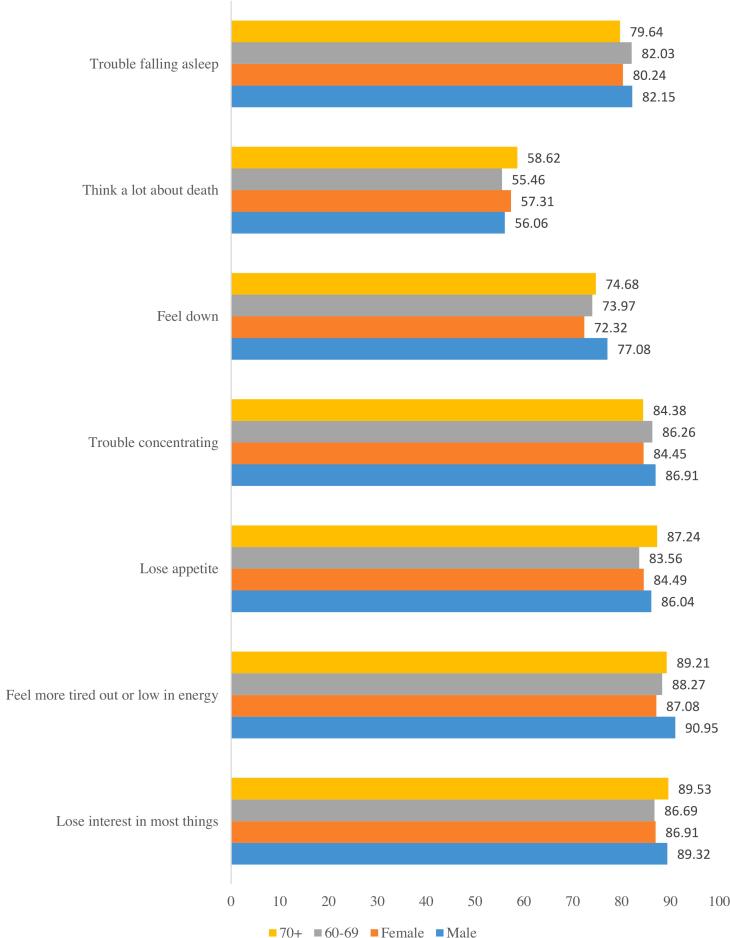
Thinking or perception during depression period among elderly

Figure 3 depicts the depression-related perception of the elderly. Almost four-fifths of the elderly male (82.15%) and female (80.24%) reported trouble falling asleep during the depression. More than half of the elderly males (56.06%) and females (57.31%) reported that they thought a lot about death during the depression. More than four-fifths of the elderly aged 60-69 years (86.26%) and elderly aged 70+ years (84.38%) had trouble concentrating during the depression. A similar percentage of elderly males (86.06%) and females (84.49%) lost appetite during the depression.

Table 3 depicts the results of the rural-urban decomposition of depression among male and female elderly. Results found that self-rated health and IADL disability explained nearly 40 percent of the urban-rural inequality in the prevalence of depression among male elderly. Tobacco use is another predictor that explained nearly 23 percent of the inequality in the prevalence of depression among the elderly. Education, self-rated health, and IADL disability were the significant predictors explaining inequality in the prevalence of depression among female elderly. Education and IADL disability explained nearly 41 percent and 47 percent of the inequality in the prevalence of depression among female elderly, respectively.

**Table 3:** Estimates of urban-rural decomposition analysis for contribution of explanatory variables for depression among male and female elderly in India.

	Male			Female		
Variables	Coefficient	Standard Error	Percent Contribution	Coefficient	Standard Error	Percent Contribution
Age	−0.0001	0.001	0.19	0.0071	0.0046	−12.93
Marital status	−0.0015	0.002	4.36	0.0024	0.0030	−4.41
Living arrangements	−0.0003	0.001	0.99	−0.0082	0.0061	14.98
Religion	−0.0015	0.001	4.25⁎⁎	−0.0023	0.0016	4.29
Education	−0.0069	0.008	20.08	−0.0220	0.0113	40.18⁎⁎
Wealth Index	−0.0004	0.001	1.26	−0.0010	0.0026	1.92
Currently working	0.0004	0.003	−1.25	0.0059	0.0058	−10.76
Self-rated health	−0.0058	0.002	17.02⁎⁎⁎	−0.0034	0.0017	6.24⁎⁎
Tobacco use	−0.0069	0.004	20.03	−0.0072	0.0062	13.24
Alcohol use	−0.0009	0.001	2.53	−0.0010	0.0029	1.89
ADL disability	−0.0017	0.001	4.90	0.0000	0.0009	0.04
IADL disability	−0.0087	0.004	25.32⁎⁎	−0.0248	0.0082	45.34⁎⁎⁎

⁎⁎ Significant at 95 percent CI.

⁎⁎⁎ Significant at 99% CI.

## 4 Discussion

Depression is a significant public health concern that is yet to be recognized as an important public health challenge. This study is very timely and significant in bringing the issue of depression among the elderly to the forefront as it is based on the nationally representative survey data. The study found that almost one-fifth (20.6%) of the elderly reported depressive symptoms. Based on 56 community-based studies in India, a meta-analysis found that the pooled prevalence of depression was 34.4 percent among elderly aged 60+ years from 1997 to 2016. A study in Bangladesh noted a higher prevalence of depression among the elderly, where almost 37 percent of the elderly reported depression [15]. Countries where the higher prevalence of depression among the elderly was reported include; 42.5 percent in Indonesia [36], 44.4 percent in Egypt [16], 23.7 percent in Thailand [50], whereas lower depression among elderly was reported in Brazil (3.8%) [7], 10.5 percent in China [13], 18.5 percent in Turkey [51]. A study based on World Health Organization (WHO) Study on Global Ageing and Adult Health (SAGE) data survey noticed that the prevalence of depression was higher in the Indian elderly than elderly in Mexico, Ghana, China, Russia, and South Africa [4]. The difference in the prevalence of depression among the elderly could be attributed to cultural differences, genetics, environmental factors, or even methodological or sampling differences [35]. However, taken together, all these studies reinforce an argument for placing greater importance on the mental health issues of older people.

This study revealed evidence of gender differences in depression among elderly people in India. The results found that the prevalence of depression was higher among elderly women than in elderly men. This finding is corroborated with the previous studies in different settings [34]. However, few studies noted an otherwise result where male elderly had a higher prevalence of depression than female elderly [32]. Worth mentioning, a few studies failed to notice any gender differences in depressive symptoms among older people [52]. Furthermore, gender differences persist with various background characteristics, disfavouring older women. The female disadvantages in depression were observed in age, living arrangement, education, wealth index, working status, self-rated health, and IADL disability, which are similar to the findings of another study in the Indian context [34]. Studies worldwide noticed that women tend to live longer than men, however, with worse health [8,30,31,40]. The fact that a larger share of Indian women than men never attended school may also partially explain the gender differences in depression [34]. Various studies have noted education as a significant predictor of depression and poor health among the elderly and other sub-populations ([11,33,[41], [42], [43]]).

The study noted male-female gradient in depressive symptoms among the elderly, where a higher proportion of elderly females reported depression than their male counterparts. However, while examining SES-related inequality in the prevalence of depression, education was a significant factor explaining the SES-related inequality in the prevalence of depression among female elderly and not in male elderly. This signifies that education predicts SES-related inequality in the prevalence of depression only among elderly females. Results also noted that a higher proportion of uneducated elderly women in rural and urban areas reported depression than highly educated elderly women in rural and urban areas. Previously available literature suggests that male-female difference in various cognition-related parameters could be controlled by education status among the elderly (P. K. [40]). Given that education inequality disfavours women in India, the study findings are not surprising at all. Better schooling during early years promote the development of brain reserve capacity, which could be a plausible explanation of higher depression level among uneducated female elderly [27]. Various studies agree with the brain reserve hypothesis and found educational attainment to be associated with various mental health issues [19,44].

Consistent with several studies in India [2,4,5,34,35,46], this study noted a higher proportion of depression among rural elderly than in urban elderly. Poor education attainment among rural people than their urban counterparts could explain the paradox of higher depression among rural elderly in parts. Better educated people tend to attain a greater sense of control, which further facilitates their adaptive strategies for coping with mental health (L. W. [29]). Lack of social support in the rural areas due to rural to urban migration of the younger population could also be a probable reason for higher depression among rural elderly in India [2]. Lack of professional health services in rural areas could also be another important factor of higher depression among rural elderly. Moreover, an underqualified and untrained medical professional in rural areas [14] could also be a significant factor for higher depression levels in rural elderly. Also, treatment-seeking is relatively lower in rural areas than in urban areas [1], which could further be attributed to higher reporting of depressive symptoms among rural elderly.

The study noted that depressive symptoms were higher among those with severe ADL and IADL disability and reported poor self-rated health. Furthermore, the study reported a significantly high SES-related inequality in the prevalence of depression among the elderly attributed to IADL disability and self-rated health. It could be inferred that IADL disability and self-rated health are the two important predictors explaining depression among the elderly. Previous research agrees with the above findings [10,25,34]. Those with poor self-rated health might perceive their health negatively, which could be a plausible explanation of higher depression. Difficulties of IADL make it tough to perform social roles and a reduced sense of mastery among older people and interrupt their independent living; all this could lead to higher depression among elderly with severe IADL [23,24].

## 5 Limitations and strengths of the study

This study has important theoretical implications in that it extends the field of research on the elderly and depression while explaining the socio-economic inequalities in the prevalence of depression. However, the study has few significant limitations. Firstly, depression was not clinically diagnosed, and it was self-reported. There are no standardized scales to capture depression or lab tests to capture medical illnesses. Depression is a social construct and may vary place to place and person to person. However, several previous studies have examined depression as a self-reported entity [10,15,26]. The self-reporting of depression could lead to overestimating the prevalence of depression and can be misleading [4]. The cross-sectional nature of data limits our understanding of causality. The reporting of depression could have been affected by the recall bias as it categorizes depression for the last 12 months. Furthermore, self-reported health can also affect the study estimates in examining the inequalities in the prevalence of depression as it was self-reported. Furthermore, cross-sectional data could have limited our understanding of causal inferences. In addition, reliability of the depression scale used for this study can be questioned and need further exploration. Despite these limitations, the study has specific strengths too. The study findings are based on a nationally representative survey released recently, thereby providing current and reliable estimates on depression among the elderly in India.

## 6 Conclusion

Unlike other previous research on the association of several risk factors with depressive symptoms among the elderly, this study focussed on the socio-economic status-related inequality in the prevalence of depression among the elderly along with focussing urban-rural and male-female gradients of depression among the elderly. The study found that almost one in every five elderly reported depression, which was higher among rural elderly and female elderly. Furthermore, self-rated health and IADL disability were the two important factors explaining the SES-related inequality in the prevalence of depression among elderly men. However, education, IADL disability, and self-rated health were three factors explaining the SES-related inequality in the prevalence of depression among elderly women.

Given the large proportion of elderly reporting depression, this study highlights the need for improving health care services among the elderly. The increasing burden of depression in specific sub-populations also highlights the importance of understanding the broader consequences of depression among rural and female elderly. Preventive measures to tackle depression among the elderly shall be considered as an integral part of public health, specifically at Primary Health Care (PHC) centers, which have a wider reach to the rural population. Furthermore, training the medical officer to identify the psychiatric problems and strengthening the national mental health program in primary health care set-up would help in early identification and appropriate management of depression among the elderly.

## Funding

This research did not receive any specific grant from funding agencies in the public, commercial, or not-for-profit sectors.

## Declaration of Competing Interest

None.
